# Is Pulmonary Mycoses Shadowed by Tuberculosis? Mandate to Hit the Bull’s Eye—An Indian Perspective

**DOI:** 10.3390/pathogens14050435

**Published:** 2025-04-30

**Authors:** Jeevarahini Regupathy, Priya Rajendran, Vinod Kumar, Sivakumar Shanmugam

**Affiliations:** 1 ICMR National Institute for Research in Tuberculosis, Chennai 600031, India; jeevarahini.r@icmr.gov.in (J.R.); shanmugam.sk@icmr.gov.in (S.S.); 2 Institute of Thoracic Medicine, Madras Medical College, Chennai 600003, India; drvinodkumar76@gmail.com

**Keywords:** tuberculosis, mycoses, fungal pathogens, misdiagnosis, co-infection, conventional methods, molecular methods

## Abstract

Tuberculosis (TB) poses serious challenges to healthcare personnel as it continues to be highly prevalent in a developing country like India. Several prominent strategies have been adopted to control this contagious infection, but the incidence rate remains high. Many studies have linked fungal infections to TB, apparently elevating concerns in the TB elimination program. Hence, it is essential to understand the mechanisms underlying TB co-infection and pulmonary mycoses to combat the problems caused by these diseases successfully. In addition, differential diagnosis of TB and fungal infections is equally essential to initiate appropriate treatment. This review attempts to describe misdiagnosis of TB and fungal disease and their relative coexistence in the context of the Indian perspective.

## 1. Introduction

Though preventable and curable, tuberculosis (TB) caused by *Mycobacterium tuberculosis* remains the second leading cause of mortality due to a single infectious agent, behind the COVID-19 infection in 2022, as stated by the WHO Global Tuberculosis Report [1]. Pulmonary TB (PTB) continues to be a serious concern in India and is consecutively listed in the top 20 TB high-burden countries despite strong measures taken by our health department [2]. Pathogenic fungi cause pulmonary mycoses via inhalation of spores, but a few fungi are endogenous, such as *Candida*, which disrupt the normal functioning of bronchial and lung tissues, resulting in pulmonary fungal disease. Furthermore, a rapid surge in fungal infection is recently attributed to other pre-existing clinical conditions such as TB, HIV/AIDS, and substantial use of immunosuppressive drugs. The compromised immune status in such circumstances allows opportunistic fungi to attack the host easily [3,4,5]. Pulmonary fungal infections are caused by *Aspergillus*, *Cryptococcus*, *Pneumocystis* [6], *Candida* [7], *Mucor* [8], and endemic fungi, which include *Blastomyces*, *Coccidioides*, and *Histoplasma* [9].

It has been reported that people recovering from TB are prone to pulmonary fungal infections and were mostly misdiagnosed as cases of relapsed PTB [10]. Another alarming fact is that there is an increase in misdiagnosis of patients with invasive fungal infections as TB patients and vice versa due to similar clinical presentations, leading to wrong or delayed treatment of the patients [11]. Therefore, studies on the simultaneous diagnosis of TB and pulmonary fungal infection and their coexistence are needed so that these patients may receive treatment with antituberculosis and/or antifungal agents promptly. To attain this, a differential diagnostic algorithm for TB and other fungal infections is required. While diagnostic methods for TB are well designed and implemented in routine programmatic setups, many labs still rely on classical diagnostic tests for fungal infections. However, these tests have very low sensitivity, with common methods involving microscopy and histopathology (79%), radiological analysis (50–90%), and culture (50–95%) [12,13]. In this review, we attempted to compile Indian studies over the past 10 years (2014–2024), focusing on the diagnosis of fungal infections with special reference to TB co-infection and misdiagnosis. We used a PubMed database for the collection of articles, using the search terms “Pulmonary infections, tuberculosis and India”, “Pulmonary mycoses, tuberculosis and India”, “Aspergillosis”, “Aspergillosis and India”, “Cryptococcosis”, “Cryptococcosis, tuberculosis and India”, “Candidiasis”, “Candidiasis, tuberculosis and India”, “Mucormycosis”, “Mucormycosis, tuberculosis and India”, “Endemic mycoses”, and “Endemic mycoses, tuberculosis and India.”

## 2. Pulmonary Fungal Infections

### 2.1. Pulmonary Aspergillosis

Pulmonary Aspergillosis can be broadly classified into three types based on clinical presentation [14].

- Allergic Bronchopulmonary Aspergillosis;
- Invasive Pulmonary Aspergillosis;
- Chronic Pulmonary Aspergillosis.

#### 2.1.1. Allergic Bronchopulmonary Aspergillosis (ABPA)

The common aetiological agent of ABPA is reported to be *A. fumigatus* [15] and is followed by other species such as *A. flavus*, *A. niger*, and *A. oryzae* [16]. The characteristic features of ABPA in conventional CT are bronchiectasis and mucoid opacities in the shape of toothpaste or a finger in a glove [17].

#### 2.1.2. Invasive Pulmonary Aspergillosis (IPA)

Inhalation of *Aspergillus* conidia results in IPA, and the subspecies of *A. fumigati* (*A. fumigatus*, *A. lentulus*, and *A. udagawae*) are involved in this common form of aspergillosis [18]. Other significant species involved in the disease are *A. flavus*, *A. nidulans*, *A. terreus*, and *A. niger.* Extensive use of corticosteroids in non-neutropenic patients eventually results in invasive aspergillosis [19]. Symptoms of IPA patients include prolonged fever, nonproductive cough, chest pain, and hemoptysis, mimicking TB [20].

#### 2.1.3. Chronic Pulmonary Aspergillosis (CPA)

The fungal pathogen causing CPA in most patients is *A. fumigatus* [21]. Incidentally, it is observed that one in five TB-negative patients after treatment with anti-tubercular therapy (ATT) might suffer from CPA [22]. A study in 2022 reported that the incidence of CPA emerging in PTB patients was estimated to be 363,601 cases and 42,766 deaths, 10.5% of total PTB deaths [23]. In addition, various studies and case reports elucidate the significance of CPA in PTB patients (Table 1). These findings indicate the importance of diagnosing CPA as part of the TB diagnostic algorithm in the program. Other predisposing factors for developing CPA into a serious illness are nontuberculous mycobacterial infections (NTM) and ABPA [24]. CPA is further subdivided into five types based on their colonization and disease development as follows:

1. Aspergillus nodules;
2. Simple Aspergilloma;
3. Chronic Cavitary Pulmonary Aspergillosis;
4. Subacute Invasive Pulmonary Aspergillosis;
5. Chronic Fibrosing Pulmonary Aspergillosis.

##### Aspergillus Nodules

Of the subtypes of CPA, *Aspergillus* nodules are relatively rare and are characterized by the CT finding of one or more nodules with or without cavities [32]. Minor or no symptoms represent this type of CPA and can only be diagnosed with histological evidence of necrosis, characterized by the presence of *Aspergillus* [33].

##### Aspergilloma

Simple Aspergilloma is indicated by a single fungal ball with radiological or microbiological evidence of *Aspergillus* species in immunocompetent individuals. Similar to *Aspergillus* nodules, this condition also accounts for minor or no symptoms [34].

##### Chronic Cavitary Pulmonary Aspergillosis (CCPA)

Complex Aspergilloma, an old surgical term now called CCPA [35], showcases parenchymal disease surrounded by a thick-walled cavity [36]. Contrary to *Aspergillus* nodules and Aspergilloma, the incidence of CCPA is high, and several reports have been documented for its role in previously treated TB patients. CT image portrays single or multiple cavities with characteristic residues in the lung [37].

##### Subacute Invasive Pulmonary Aspergillosis (SAIA)

Occasionally called semi-IPA and chronic necrotizing pulmonary aspergillosis [38], SAIA is similar to CCPA in radiology, with only the distinction of rapid progress under the former condition. Clinical conditions that make the host vulnerable to SAIA are diabetes, malnutrition, corticosteroid use, connective tissue disorders, HIV infection, and COPD [39].

##### Chronic Fibrosing Pulmonary Aspergillosis (CFPA)

The final stage of CPA is expressed as CFPA, which is more commonly reported along with CCPA than other types of CPA [40]. Since this condition overlaps with different forms of CPA, a distinctive diagnosis is impossible [24].

### 2.2. Pulmonary Cryptococcosis

Though 30 species of *Cryptococcus* are identified, *C. neoformans* and *C. gattii* are known to cause human infections [41]. Invasive fungal diseases in immunocompromised individuals are known to be caused by *C. deneoformans* [42], while *C. gattii* causes infection in immunocompetent individuals [43]. CT findings of pulmonary cryptococcosis (PC) include pulmonary nodules, patchy shadows, ground-glass attenuation, interstitial changes, and cavitation. In addition, the left lower lung seems to be involved most frequently in immunocompromised patients and is prone to being misdiagnosed as TB or a tumor [44,45,46]. Although not common, studies from various parts of India have been reporting the co-infection of TB with cryptococcosis, emphasizing routine monitoring (Table 2).

### 2.3. Pneumocystis Pneumonia

Pneumocystis pneumonia (PCP) is caused by an opportunistic pathogen named *Pneumocystis jirovecii* [52]. It is an ascomycete fungus that was previously categorized as a protozoan due to its similarity in morphology and drug susceptibility [53] but later as a fungus based on RNA analysis [54]. The clinical manifestations of PCP are illustrated by the subacute onset of dyspnoea, nonproductive cough, and low-grade fever [55]. Few studies and case reports have been recorded on pneumocystis pneumonia and TB as opportunistic infections in HIV populations across India, but none involved a co-infection or misdiagnosis [56,57]. This could be due to its coexistence in the HIV population, where the patient is diagnosed with one of the diseases, and by the time the other one is suspected, the patient dies [58].

### 2.4. Pulmonary Blastomycosis

The inhalation of *Blastomyces* spores results in pulmonary disease, and the species involved are *Blastomyces dermatitidis*, *B. percursus*, and *B. emzantsi* [59]. Although the lung is considered a common site of infection [60], the first human case of this infection was documented in the skin tissue [61]. This extrapulmonary spread could be attributed to a hematogenous initial lung infection [62]. The pulmonary form of the disease is expressed as pneumonia with an insidious course, while chest CT is non-specific, and infection limited to the lungs can mimic *M. tuberculosis* [63,64]. Blastomycosis misdiagnosed as TB was reported from Kerala, India, in 2019. The patient was on empirical ATT for nearly a year due to an indefinite diagnosis. History of his travels to Chicago, an endemic region for Blastomycosis, stirred suspicion and was he appropriately diagnosed [65].

### 2.5. Pulmonary Coccidioidomycosis

Arthroconidia of the fungi *Coccidioides immitis* and *Coccidioides posadasii*, usually present in the soil, are responsible for pulmonary coccidioidomycosis [66]. The disease is reported very rarely in places other than endemic areas [67] and is commonly found in association with human and animal middens [68]. Though endemic to deserts in the United States, as well as Central and South America, travel history to these countries has resulted in cases reported from other parts of the world [69]. It has been documented that about 19.5% of Coccidioidomycosis-confirmed cases involved TB as well [70]. One case report has been documented so far from India, where the diseased individual with a travel history presented with complaints of swelling over the neck and was clinically diagnosed as having TB. Further diagnosis by fungal stains, fungal culture, and serology confirmed *Coccidioides*, and a course of fluconazole was used for treatment [71].

### 2.6. Pulmonary Histoplasmosis

*Histoplasma capsulatum*, a dimorphic fungus, is responsible for establishing histoplasmosis infection [72]. Disease transmission is reported to be due to inhalation of spores in instances of soil disruption like cave excavation [73]. Chronic histoplasmosis is often characterized by nodule formation, and this clinical condition is termed chronic cavitary pulmonary histoplasmosis (CCPH) [74]. Major clinical features of TB, like radiological findings, pulmonary infiltrates, hilar lymphadenopathy, opacities of the lung, and pleural effusion, are also reported in histoplasmosis [75,76]. Instances of histoplasmosis misdiagnosed as TB across India have been recorded in Table 3.

### 2.7. Pulmonary Candidiasis

Opportunistic candidal infection is caused frequently by *Candida albicans*, although the incidence of disease by other members of the same genus is also increasing rapidly [81]. *Candida*, a commensal of the gut microbiome, shapes itself into a pathogen based on antibiotics, with severe lapse leading to compromise of the gut integrity, host immune dysfunction, use of central venous catheter, surgery, etc. [82]. Diagnosing pulmonary candidiasis is tedious, as no specific clinical and radiographical presentations exist. Hence, pulmonary parenchymal invasion through histopathologic examination can be a more reliable method for diagnosis of patients with deep-rooted invasion, though such instances occur rarely [83]. A systematic review and meta-analysis of different cross-sectional studies by Fishani et al. has established a significantly higher incidence of co-infection of candidiasis among PTB patients in Asia and Africa [84]. Significant correlations between HIV, TB, and candidiasis that were documented from India are included in Table 4.

### 2.8. Pulmonary Mucormycosis

The order Mucorales includes different genera, such as *Rhizopus*, *Mucor*, *Lichtheimia*, *Rhizomucor*, *Cunninghamella*, and *Apophysomyces* spp., and are involved in establishing the invasive fungal infection called mucormycosis [87]. Diabetes mellitus, solid organ transplant, chronic renal failure, hematological malignancy, acute myeloid leukemia, and TB are the predisposing risk factors for mucormycosis [88]. Clinical features include ground-glass lesions, reverse halo signs, necrosis, lobar and segmental consolidation, and single or multiple lesions [89]. Studies and case reports on mucormycosis documented across India have been described in Table 5.

## 3. Diagnosis

**Figure 1:**
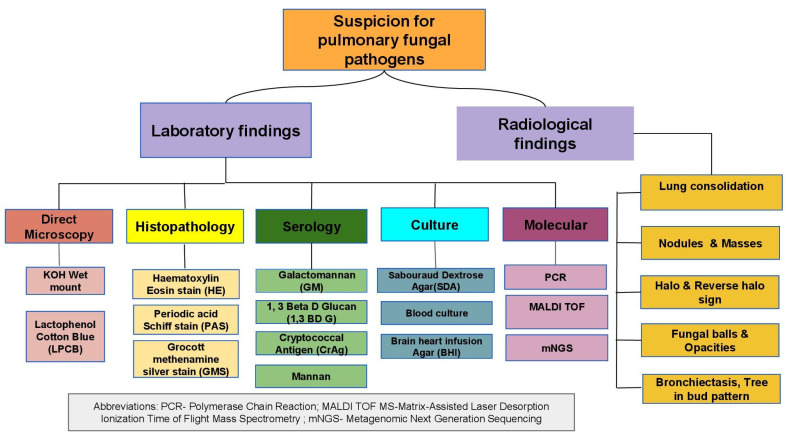
Diagnostic tools for fungal infections in patients with pulmonary diseases.

The Fungal Priority Pathogen List released by WHO in 2022 underscores the need for quick measures to prevent, diagnose, and appropriately treat fungal pathogens [93]. Some of the proven diagnostic tools available for detecting pulmonary mycoses can be broadly categorized into histopathological and culture methods, while radiological, immunological, and molecular techniques are considered for probable diagnosis [94]. Figure 1 shows diagnostic techniques for suspected pulmonary fungal infections in presumed TB patients who have been microbiologically determined to be non-TB patients, with confirmed possibility of bacterial and viral pneumonias ruled out.

### 3.1. Direct Microscopy and Histopathological Examination

Histopathological examination can be carried out by staining methods such as Hematoxylin and Eosin staining (HE), periodic acid Schiff staining (PAS), or Gomori’s methenamine silver staining (GMS) [95]. An evaluation study carried out to detect fungal infections in post-COVID-19 patients by Baxi et al. from Gujarat compared the efficacies of histopathological, culture, and KOH wet mount methods, of which the histopathological diagnosis by HE staining was found to be the most sensitive and rapid technique for mycological identification [96]. Another retrospective study from a tertiary care hospital in Jammu and Kashmir stressed the potential misdiagnosis in the microbiological examination, as it cannot differentiate between pathogenic fungi and contamination. Furthermore, they suggested that histopathology could be more reliable for fungal diagnosis as it can distinguish between contamination, colonization, or actual infection [97]. Seth et al. from AIIMS, New Delhi, reviewed the diagnostic progress of fungal infections and concluded that diagnostic tools need to be validated in younger children. They also admitted that histopathological examination remains the gold standard, while galactomannan assay or PCR techniques can be employed for pediatric populations [98].

### 3.2. Radiology

Radiological diagnosis includes the utility of X-rays, CT scans, radiofrequency (MRI), and sound waves (ultrasound) for infection identification [99]. High-Resolution Computed Tomography (HRCT), though insensitive, can be utilized as a screening tool initially in highly suspected cases, and an empirical therapy could be started when microbiological diagnosis takes up more time [100]. It has been documented that PJP by *Pneumocystis* in HIV/AIDS patients can be suspected with radiological evidence. Similarly, lung consolidation, nodules, and masses can be used to suspect cryptococcosis and blastomycosis [101]. Garg et al. from PGIMER, Chandigarh, studied the imaging spectrum of CPA and summarized that a patient’s immune status plays a significant role in radiological diagnosis, as clinical presentation is often not specific and leads to misdiagnosis [39].

### 3.3. Culture

Sabouraud’s Dextrose agar, Potato Dextrose agar with certain antibiotics, or Brain Heart Infusion agar enriched with blood is generally used for growing fungal pathogens [102]. The culture of clinical specimens is considered more efficient in determining the specific fungal etiological agent if the sample is positive, and susceptibility patterns can be determined. When culture is challenging to identify using tease mounts, the slide culture technique has to be used alternately. Furthermore, the appropriate specimens from the lower respiratory tract for fungal diagnosis, as stated by the ICMR SOP for Fungal Identification and Detection of Antifungal Resistance, are sputum, bronchoalveolar lavage (BAL), and lung biopsy. The collection of these specimens should be carried out in a defined manner for accurate results. Sputum should be collected in the early morning after mouth rinsing and brushing, with an adequate volume range is 5–10 mL. BAL samples must be collected in a sterile container using fiberoptic bronchoscopes. Lung biopsy samples can be taken through fluoroscopy-guided trans-thoracic needle aspiration or open lung biopsy. However, despite open lung biopsy being the best method, it has high safety concerns and needs a highly trained professional for specimen collection [103].

### 3.4. Serology

Serum galactomannan (GM) testing can be recommended for IPA suspects initially if there is still high clinical suspicion for IPA, and they can be subjected to tissue biopsy only when a GM negative result is obtained. Consequently, false-positive serum GM can be avoided with a BAL GM confirmation [104]. Another biomarker, β-D-glucan, is found in the cell wall of most fungi, with the exceptions of *Cryptococcus neoformans* and *Mucormycetes* [105]. However, in the case of ABPA, *Aspergillus*-specific IgG has proven to have higher sensitivity and specificity than IgE [106], GM, and B, D-glucan [107]. Other serological assays with significant diagnostic ability for fungal detection include immunodiffusion (ID), counter-immunoelectrophoresis (CIE), enzyme-linked immunosorbent assays (ELISAs), complement fixation (CF), radio-immunosorbent assays (RIAs), and agglutination assays [108].

### 3.5. Molecular Diagnosis

Gudisa et al. from PGIMER, Chandigarh, reviewed the evolution of molecular diagnostics for fungal infection over the years and elucidated the significance of RT-PCR, the role of Sanger sequencing, and next-generation sequencing in the detection and speciation of fungal pathogens [109]. The review article described the utility of different molecular methods, including PCR, loop-mediated isothermal amplification, nucleic acid sequence-based amplification, and rolling circle amplification in the diagnosis of fungal infections. Although PCR has been widely used, the role of other molecular techniques in diagnosing pulmonary disease has been less explored and needs further research in India. A study from North India by Srinivas et al. from Maharashtra evaluated GenoSen’s panfungal RT PCR kit. It depicted the utility of panfungal PCR in a resource-limited country like India [110].

#### 3.5.1. Metagenomic Next-Generation Sequencing (mNGS)

Molecular methods such as mNGS are fast-growing diagnostic techniques with the subsequent advantage of rapid and broad detection [111]. The technique will greatly help in differential diagnosis but requires strong validation and standardization for better utility in metagenomic next-generation sequencing [112]. Different studies have demonstrated the efficiency of the NGS technique in detecting fungal pathogens such as *Pneumocystis*, *Histoplasma*, *Aspergillus*, and *Candida* [113].

#### 3.5.2. Matrix-Assisted Laser Desorption–Ionization Time of Flight Mass Spectrometry (MALDI-TOF MS)

Ghosh et al. tried to evaluate the utility of MALDI-TOF MS in diagnosing bloodstream yeasts. They also added the need for extensive studies to be carried out to validate the potential of MALDI-TOF as a diagnostic technique for effective patient care and management [114]. Paul et al. standardized the MALDI-TOF method with different protocols and concluded it could be used for routine diagnosis of pathogenic molds [115].

### 3.6. Limitations of Fungal Diagnosis

For most of the fungal infections, conventional methods (microscopy, histopathology, and culture) remain the gold standard of diagnosis, but the diagnostic yield of culture is very low, and the time to positivity is higher. Furthermore, histopathology requires highly trained personnel to identify the pathogen accurately. Serological assays are promising, but a major setback is their cross-reactivity and lower sensitivity. There have been significant advances in the development of PCR assays for fungal diagnosis in recent years, and their utility has been approved by the European Organization for Research and Treatment of Cancer (EORTC). But one of the drawbacks of fungal PCR is its inability to distinguish airway colonization from an infection [7].

## 4. Key Points

1. Clinical suspicion of fungal pathogens in ATT non-responders should be considered before treating them further.
2. Early differentiation of active PTB, PTLD, and PTB from fungal co-infection in settings with a high TB burden is the need of the hour.
3. A broader screening strategy at the investigation stage itself should be proposed.
4. Microbiological confirmation with an inclusion of simultaneous fungal and TB diagnostic methods for presumptive PTB patients with abnormal chest X-ray will facilitate appropriate diagnosis and treatment.

## 5. Conclusions

In this review article, we aimed to elucidate the significance of pulmonary mycoses being misdiagnosed or ignored in the context of TB. It is prominent that the incidence of misdiagnosis or no diagnosis of pulmonary mycosis among TB patients is common. Although some studies have documented this scenario, more instances of this could be anticipated without being reported. Hence, clinical suspicion of pulmonary mycoses among TB patients and ATT non-responders could help in timely prognosis and patient care. Further, it emphasizes the urgent necessity to develop differential diagnostics for TB and pulmonary mycoses, as most of the available tools have been exposed to either cross-reactivity or low specificity.

## Figures and Tables

**Table 1:** Studies and case reports involving Pulmonary Aspergillosis and PTB.

Type of Aspergillosis	Reported State and Year	Type of Study and Objectives	Study Population	Number of Isolates	Salient Findings	Reference
Chronic Pulmonary Aspergillosis (CPA)	New Delhi 2024	Prospective Objective: To confirm the presence of CPA in newly diagnosed PTB at baseline and the end of TB therapy	255—Recruited 158—Completed follow-up	11.1% were positive at baseline, and 27.5% were positive at the end of anti-tubercular therapy	CPA can arise as Post-TB Lung Disease (PTLD) or exist as a co-infection with TB in new patients.	Jha et al. [25]
Chronic Pulmonary Aspergillosis	Maharashtra 2023	Cross-sectional/Observational Objective: To determine the prevalence of CPA in patients with treated fibrocavitary PTB	42	9.5%	Serological diagnosis is necessary for detecting CPA in patients with or without TB due to similar clinical features.	Rajpurohit et al. [26]
Chronic Pulmonary Aspergillosis	New Delhi 2022	Prospective-Observational Objective: To investigate the diagnostic accuracy, sensitivity, and specificity of different computed tomography (CT) results in identifying recurrence in suspects of PTB.	130	24.2%	The mediastinal necrotic lymph node is the appropriate CT finding for differentiating between recurrent TB and post-TB sequelae in CPA complications.	Bharath et al. [27]
Chronic Pulmonary Aspergillosis	India 2022	Estimation Analysis Objective: To estimate the prevalence of CPA related to PTB.	-	-	Comprehensive estimation of total CPA burden in pulmonary TB patients	Denning et al. [23]
Chronic Pulmonary Aspergillosis	Chandigarh 2023	Retrospective Objective: To determine the role of C-reactive protein (CRP) and erythrocyte sedimentation rate (ESR) for diagnosing chronic pulmonary aspergillosis (CPA)	434 subjects and 20 disease controls	-	Erythrocyte Sedimentation rate (ESR) and C-reactive protein (CRP) play a significant role as biomarkers in CPA diagnosis among Post-TB Lung Disease (PTLD) cases.	Sehgal et al. [28]
Aspergilloma	Uttarakhand 2023	Case Report	-	1	Aspergilloma was found as a mass in the intrabronchial region instead of a lung cavity in a patient with TB a decade ago.	Lahiri et al. [29]
Invasive Pulmonary Aspergillosis	Chandigarh 2022	Case Report	-	1	A 47-year-old male had a sequelae of Coronavirus Disease-2019 (COVID-19) complicated by TB and IPA	Gandotra et al. [30]
Chronic Cavitary Pulmonary Aspergillosis	Karnataka 2022	Case Report	-	1	In a 57-year-old adult male, COVID-19 had reactivated latent aspergilloma, and the condition developed into CCPA, a more severe form of aspergillosis. This patient reportedly had a TB and aspergilloma co-infection 20 years ago.	Chaurasia et al. [31]

**Table 2:** Case reports on pulmonary cryptococcosis and PTB.

Reported State/Year	Site of Infection	Salient Findings	Reference
New Delhi 2019	Lung	A 26-year-old female was initially diagnosed with TB, and treatment was initiated, but she was later diagnosed with cryptococcosis.	Meena et al. [47]
New Delhi 2018	Lung and CNS	An immunocompetent child suspected of having TB was diagnosed with disseminated cryptococcosis at a later stage	Ismail et al. [48]
New Delhi 2016	Lung	In a 36-year-old male, co-infection of pulmonary cryptococcosis and TB was reported.	Jain et al. [49]
New Delhi 2016	Lung	A 45-year-old male with a history of TB was presented with a mass in the right lower lobe; similar to a lung tumor, but was eventually diagnosed as cryptococcoma through CT and histopathological examination.	Pawar et al. [50]
New Delhi 2015	Lung/Adrenal gland	A 45-year-old male, with no TB history but initially diagnosed as disseminated TB, was later diagnosed with cryptococcosis by histopathological findings.	Ranjan et al. [51]

**Table 3:** Case reports on pulmonary histoplasmosis being misdiagnosed as TB.

Reported State/Year	Site of Infection	Salient Findings	Reference
Rajasthan 2021	Lung	A 28-year-old with pulmonary histoplasmosis was misdiagnosed as miliary TB	Agarwal et al. [77]
Chandigarh 2020	Skin, Lung	Disseminated TB and histoplasmosis co-infection were reported in a 50-year-old male.	Anot et al. [78]
New Delhi 2019	Lung	Multi-Drug Resistant-TB was suspected in a 59-year-old female with a history of cutaneous TB who presented with manifestations such as fever and dry cough. Eventually, a PET scan revealed a soft tissue nodule, and histopathological examination confirmed the presence of *H. capsulatum*.	Dutta et al. [79]
Telangana 2018	Adrenal gland, Lung	Hepatitis C was confirmed; the case study presented with fever and was treated for four months with ATT for suspected TB, but was finally diagnosed with Histoplasmosis, which mimicked TB in clinical manifestations.	Ramesh et al. [80]

**Table 4:** Studies on pulmonary candidiasis and TB co-infections across India.

Reported State/Year	Type of Study	Study Population	Number of Isolates	Site of Infection	Salient Findings	Reference
Uttarakhand 2016	Case Report	-	1	Lung	In a 30-year-old female, the coexistence of drug-resistant TB with invasive candidiasis was reported.	Khanduri et al. [85]
Rajasthan 2016	Prevalence Study Objective: To determine the prevalence of opportunistic candidal infection in TB patients	60 confirmed Pulmonary TB patients	33	Lung	The presence of Candida in sputum samples among PTB patients was reported using SDA and ChromAgar cultures.	Astekar et al. [81]
Maharashtra 2016	Prospective Observational Study Objective: To study the clinical profile of renal transplant recipients.	45 renal transplant recipients (RTRs)	7	-	Among renal transplant recipients, TB with candidiasis and CMV with TB were found in 7 patients.	Kumar et al. [86]

**Table 5:** Case reports on pulmonary mucormycosis and TB.

State	Year	Site of Infection	Salient Findings	Reference
Chandigarh 2015	2015	Lung	Co-infection of TB and mucormycosis in a 30-year-old diabetic patient was reported.	Aggarwal et al. [90]
Puducherry 2016	2016	Lung	Pulmonary TB with mucormycosis co-infection was demonstrated in a 72-year-old diabetic patient admitted to the intensive care Unit.	Dube et al. [91]
New Delhi 2020	2020	Lung	Disseminated pulmonary mucormycosis and TB co-infection in a diabetic patient was reported.	Ramesh et al. [92]
